# Prognostic value of pre-treatment [^18^F] FDG PET/CT in recurrent nasopharyngeal carcinoma without distant metastasis

**DOI:** 10.1186/s12885-024-12189-7

**Published:** 2024-04-15

**Authors:** Zhe Dong, Gao-Yuan Wang, Dong-Yu Dai, Guan-Jie Qin, Ling-Long Tang, Cheng Xu, Jun Ma

**Affiliations:** Department of Radiation Oncology, State Key Laboratory of Oncology in South China, Guangdong Key Laboratory of Nasopharyngeal Carcinoma Diagnosis and Therapy, Guangdong Provincial Clinical Research Center for Cancer, Sun Yat-sen University Cancer Center, 510060 Guangzhou, P. R. China

**Keywords:** Nasopharyngeal carcinoma, [18F] FDG PET/CT, TNM, Locoregional recurrent

## Abstract

**Background:**

[18 F]-Fluorodeoxyglucose (FDG) positron emission tomography/computed tomography (PET/CT) has the ability to detect local and/or regional recurrence as well as distant metastasis. We aimed to evaluate the prognosis value of PET/CT in locoregional recurrent nasopharyngeal (lrNPC).

**Methods:**

A total of 451 eligible patients diagnosed with recurrent I-IVA (rI-IVA) NPC between April 2009 and December 2015 were retrospectively included in this study. The differences in overall survival (OS) of lrNPC patients with and without PET/CT were compared in the I-II, III-IVA, r0-II, and rIII-IVA cohorts, which were grouped by initial staging and recurrent staging (according to MRI).

**Results:**

In the III-IVA and rIII-IVA NPC patients, with PET/CT exhibited significantly higher OS rates in the univariate analysis (*P* = 0.045; *P* = 0.009; respectively). Multivariate analysis revealed that with PET/CT was an independent predictor of OS in the rIII-IVA cohort (hazard ratio [HR] = 0.476; 95% confidence interval [CI]: 0.267 to 0.847; *P* = 0.012). In the rIII-IVA NPC, patients receiving PET/CT sacns before salvage surgery had a better prognosis compared with MRI alone (*P* = 0.036). The recurrent stage (based on PET/CT) was an independent predictor of OS. (r0-II versus [vs]. rIII-IVA; HR = 0.376; 95% CI: 0.150 to 0.938; *P* = 0.036).

**Conclusion:**

The present study showed that with PET/CT could improve overall survival for rIII-IVA NPC patients. PET/CT appears to be an effective method for assessing rTNM staging.

**Supplementary Information:**

The online version contains supplementary material available at 10.1186/s12885-024-12189-7.

## Background

Nasopharyngeal carcinoma (NPC), arising from the squamous cells of epithelial lining in the nasopharynx, has an extremely marked geographical variations which was prevalent in Southern China [1, 2]. The crude incidence rate of NPC has reached 1.5 per 100,000 individuals, as reported by Globocan 2018 [3]. Over the years, advancements in treatment modalities, such as magnetic resonance imaging (MRI), intensity-modulated radiotherapy (IMRT), and induction chemotherapy (IC), have contributed to improved outcomes for NPC patients, with 5-year survival rates ranging from 85 to 90% [1, 4]. However, a subset of patients, approximately 10–20%, still experience locoregional tumor residues or recurrence [1, 4]. Therefore, it’s crucial to implement effective routine examinations for local and/or regional surveillance that can detect recurrence at an earlier stage and provide valuable guidance for treatment decisions [5].

MRI is the primary method used for routine monitoring of locoregional recurrence nasopharyngeal carcinoma (lrNPC) due to its high anatomical resolution and ability to provide detailed soft tissue contrast images [6, 7]. It can also provide accurate diagnosis, staging, and guidance for treatment of recurrence [7, 8]. Notably, [18 F]-Fluorodeoxyglucose (FDG) positron emission tomography/computed tomography (PET/CT) is highly recommended as a systemic diagnostic tool in NPC due to its superior ability to detect both primary and/or regional recurrence as well as distant metastasis [9–11]. It is undeniable that [18 F]-FDG PET/CT is unable to detect skull base involvement and intracranial extension, as well as brown fat throughout the head and neck region, which may be one of the reasons why radiotherapists prefer MRI for staging and target area delineation in the initial diagnosis of nasopharyngeal carcinoma in clinical practice [12–14]. However, several studies have demonstrated that PET/CT has higher sensitivity and specificity compared to MRI in the diagnosis of local and lymph node recurrence in NPC at recurrence [15–17]. A meta-analysis by Li et al. showed that patients MRI versus (vs.). PET-CT had lower sensitivity (0.83 vs. 0.92) and specificity (0.78 vs. 0.89) in diagnosis of local recurrence and residue of NPC after IMRT [16]. Similarly, OuYang et al. reported that PET/CT vs. MRI had a higher sensitivity in detecting local (93.9% vs.79.3%; *P* < 0.001) and lymph nodes (90.9% vs. 67.6%; *P* < 0.001) recurrence and achieved more accurately recurrence staging (rStage) in NPC [17]. Altogether, it indicated that PET/CT can provide more accurate imaging for lrNPC. However, as an expensive examination, it is unclear whether PET/CT is necessary to perform after MRI or other imaging modalities have already detected the tumor with locoregional recurrence. In other words, it is unknown whether the advantages of PET/CT can translate into improved prognosis for lrNPC patients. Therefore, the aim of this study was to evaluate the prognostic value of PET/CT in lrNPC.

## Materials and methods

### Patients

**Fig. 1:**
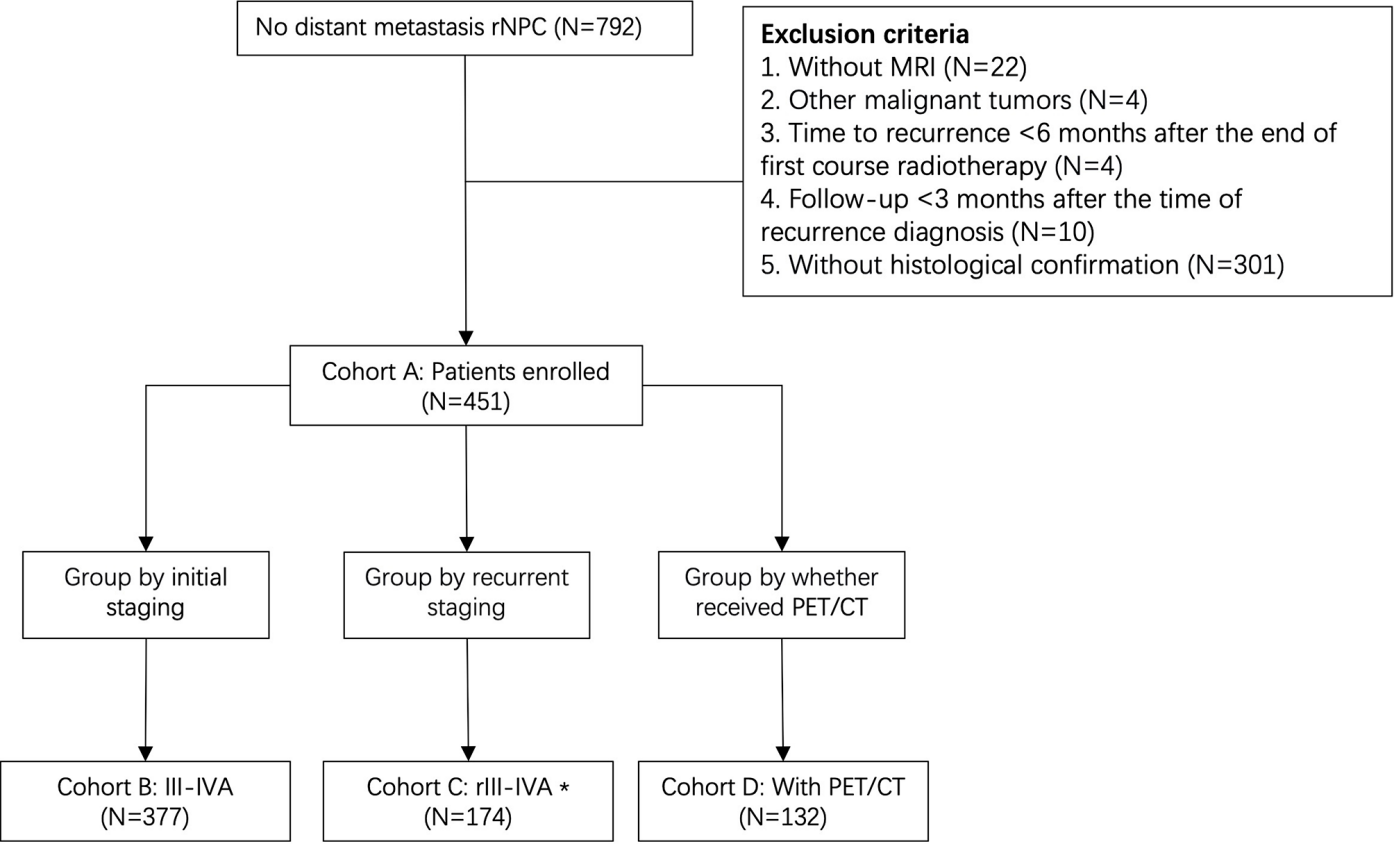
Flow diagram of the study. Cohort B (III-IVA), Cohort C (rIII-IVA), and Cohort D (with PET/CT) were grouped according to initial staging, recurrent staging, and whether PET/CT was performed at the time of recurrence detection, respectively. Abbreviations: r: Recurrent; N: number; NPC: nasopharyngeal carcinoma; MRI: magnetic resonance imaging; PET/CT: positron emission tomography/computed tomography. *Based on the eighth edition of the Union for International Cancer Control/American Joint Committee on Cancer (UICC/AJCC) staging system according to MRI

A total of 451 eligible patients diagnosed with recurrent I-IVA nasopharyngeal carcinoma (NPC) between April 2009 and December 2015 were retrospectively identified from the NPC-specific database at the Sun Yat-sen University Cancer Center, which included a total of 10,126 patients. Inclusion criteria: (1) pathologically confirmed as NPC; (2) with MRI; (3) without distant metastasis at initial diagnosis and first recurrence; (4) confirmed with recurrent NPC with regular follow-up > 6 months after the end of first course radiotherapy. Exclusion criteria: (1) other malignant tumors; (2) insufficient monitoring data; (3) pregnancy or lactation. We then screened out Cohort B (III-IVA), Cohort C (rIII-IVA), and Cohort D (with PET/CT) based on eligible patients’ initial staging, recurrent staging (according to MRI), and whether PET/CT was performed at the time of recurrence detection, respectively (Fig. 1). The study protocol was approved by the committees of the Institutional Review Boards at Sun Yat-sen University Cancer Center.

### Clinical characteristics

All clinical characteristics were obtained prior to retreatment for recurrent NPC. Each patient underwent following evaluations: physical examination, head and neck magnetic resonance imaging, and biopsy of the nasopharynx or cervical lymph nodes. Additionally, we collected the features of PET-CT, biopsy pathology, and cervical sonography within two months at the time of performing MRI. Blood examination variables before recurrent treatment included lactate dehydrogenase (LDH), C-reactive protein (rCRP), albumin (rALB), lymphocyte counts, neutrophil counts, and plasma Epstein-Barr virus (rEBV) DNA loads. Other demographic information of the patients was collected, including age, gender, history of re-radiotherapy, surgery, chemotherapy, initial T stage, recurrent T stage (rT stage), initial N stage, and recurrent N stage (rN stage).

### MRI and PET/CT protocol

MR images of the nasopharynx and neck regions were assessed using a 1.5-T or 3.0-T system prior to retreatment initiation. The evaluation included non-enhanced fast spin-echo (FSE) and enhanced fat-suppressed T1-weighted images (T1WIs) with a repetition time (TR) of 500–550 ms and an echo time (TE) of 10–15 ms on the axial, sagittal, and coronal planes. Additionally, all patients underwent T2-weighted FSE sequences with a TR of 4000–5500 ms and a TE of 90–110 ms in the axial plane. The scanning section thicknesses were 5 mm for the axial plane and 2–3 mm for the sagittal and coronal planes.

[18 F] FDG PET/CT images were acquired using the PET/CT scanner (Discovery ST, GE Healthcare, Waukesha, WI; or Biograph mCT, Siemens Healthcare, Erlangen, Germany) following PET/CT guidelines for tumor imaging [18]. Prior to the PET scan, a low-dose CT scan was performed. For the Discovery ST scanner, the CT scan parameters were as follows: automatic tube current modulation, tube voltage of 140 kV, collimation of 16 × 1.25 mm, rotation time of 0.8 s, and a slice thickness of 3.75 mm. For the Biograph mCT scanner, the CT scan parameters were as follows: tube current of 80–200 mAs, voltage of 120 kV, collimation of 32 × 1.25 mm, rotation time of 0.5s, and a slice thickness of 3 mm. Subsequent emission images were acquired for 3 min per bed position in two dimensions (2D) for the Discovery ST scanner or 1.5–2 min per bed in three dimensions (3D) with 6 to 8 beds for the Biograph mCT scanner. Before the injection of [18 F] FDG, patients fasted for over 6 h to control blood glucose levels. The patients were then injected with a specific dose of [18 F] FDG based on their body weight. Imaging was performed 55–80 min after the injection. PET/CT images were reconstructed using an ordered subset expectation maximization iterative image reconstruction method, and the slice thickness was 2 mm (3D) in a 200 × 200 matrix or 3.25 mm (2D) in a 128 × 128 matrix. And Chest radiography/computed tomography (CT), skeletal scintigraphy, and abdominal sonography/CT were performed to rule out distant metastasis.

### Follow-up and outcomes

The primary endpoint was overall survival (OS) defined as freedom from the date of recurrent NPC diagnosis to the death due to any cause. Patients were followed every 3 months during the first 2 years after retreatment, then once every 6 months or until death. During follow-up period, various diagnostic procedures were conducted, including plasma EBV-DNA screening, physical examination, abdominal ultrasound, chest X-ray, and MRI of the nasopharynx and neck. For advanced or suspected distant metastasis, PET/CT or skeletal scintigraphy was recommended, and biopsies were performed if necessary.

### Statistical analysis

The optimal cutoff value of LDH was achieved according to previous study, and of other continuous variables were determined by maximally selected rank statistics method with the “maxstat” package (Fig.S1) [19, 20]. The unadjusted actuarial rates were estimated using the Kaplan-Meier method, and differences between groups were assessed using the log-rank test. Multivariate analyses were conducted using the Cox proportional hazards model to calculate hazard ratios with a 95% confidence level (CI). Statistical significance was defined as two-tailed *P*-values < 0.05. All statistical analyses were performed using SPSS version 27.0 (IBM Corporation, Armonk, NY) and R (version 4.2.1) (http://www.r-project.org).

## Results

### Patients

A total of 451 NPC patients were enrolled in the study, with a median follow-up period of 32.6 months (interquartile range [IQR]: 15.8–47.2 months) and 164/451 (36.3%) patients died. The OS rates at 1, 3, and 5 years were 89.7%, 66.8%, and 52.4%, respectively. Baseline characteristics were shown in Table 1. Among these patients, 377 (83.6%), 174 (38.6%), and 132 (29.3%) were III-IVA stage, rIII-IVA stage, and with PET/CT, respectively (Fig. 1). After diagnostic recurrence, 29/451 (6.4%) patients experienced distant metastases (including 6 cases of lung, 9 cases of liver, 5 cases of bone, 1 case of mediastinal, and 8 cases of two or more sites).

**Table 1:** Patient characteristics

Characteristics	With PET/CT N = 132 (%)	Without PET/CT N = 319 (%)
Age (years)		
< 45	65 (49.2%)	169 (53.0%)
≥ 45	67 (50.8%)	150 (47.0%)
Sex		
Male	106 (80.3%)	238 (74.6%)
Female	26 (19.7%)	81 (25.4%)
rEBV DNA (copies/ml)		
Undetectable	48 (36.4%)	105 (32.9%)
Detectable	69 (52.3%)	167 (52.4%)
Unkown	15 (11.4%)	47 (14.7%)
rLactate dehydrogenase (U/L)		
< 240	117 (88.6%)	247 (77.4%)
≥ 240	5 (3.8%)	7 (2.2%)
Unkown	10 (7.6%)	65 (20.4%)
rC-reactive protein (mg/L)		
< 10.5	107 (81.1%)	218 (68.3%)
≥ 10.5	18 (13.6%)	30 (9.4%)
Unkown	7 (5.3%)	71 (22.3%)
rAlbumin (g/L)		
<39.4	4 (3.0%)	30 (9.4%)
≥ 39.4	121 (91.7%)	224 (70.2%)
Unkown	7 (5.3%)	65 (20.4%)
rNLR		
< 3.7	89 (67.4%)	189 (59.2%)
≥ 3.7	41 (31.1%)	108 (33.9%)
Unkown	2 (1.5%)	22 (6.9%)
rT category*		
rT0-rT2	91 (68.9%)	228 (71.5%)
rT3-rT4	41 (31.1%)	91 (28.5%)
rN category*		
rN0	80 (60.6%)	159 (49.8%)
rN1-rN3	52 (39.4%)	160 (50.2%)
rStage*		
r0-rII	78 (59.1%)	199 (62.4%)
rIII-rIVA	54 (40.9%)	120 (37.6%)
Intial stage		
I-II	23 (17.4%)	51 (16.0%)
III-IVA	109 (82.6%)	268 (84.0%)
Pathological examination sites		
Primary tumor	79 (59.8%)	172 (53.9%)
Regional lymph nodes	50 (37.9%)	138 (43.3%)
Both	3 (2.3%)	9 (2.8%)
Treatment		
Salvage Surgery	60 (45.5%)	160 (50.2%)
Re-radiotherapy	48 (36.4%)	84 (26.3%)
Palliative treatment	24 (18.2%)	75 (23.5%)

Abbreviations: r: Recurrent; EBV: epstein-barr virus; NLR: neutrophil-to-lymphocyte ratio; PET/CT: positron emission tomography/computed tomography

* Based on the eighth edition of the Union for International Cancer Control/American Joint Committee on Cancer (UICC/AJCC) according to MRI

### With PET/CT vs. MRI alone in prognosis

**Fig. 2:**
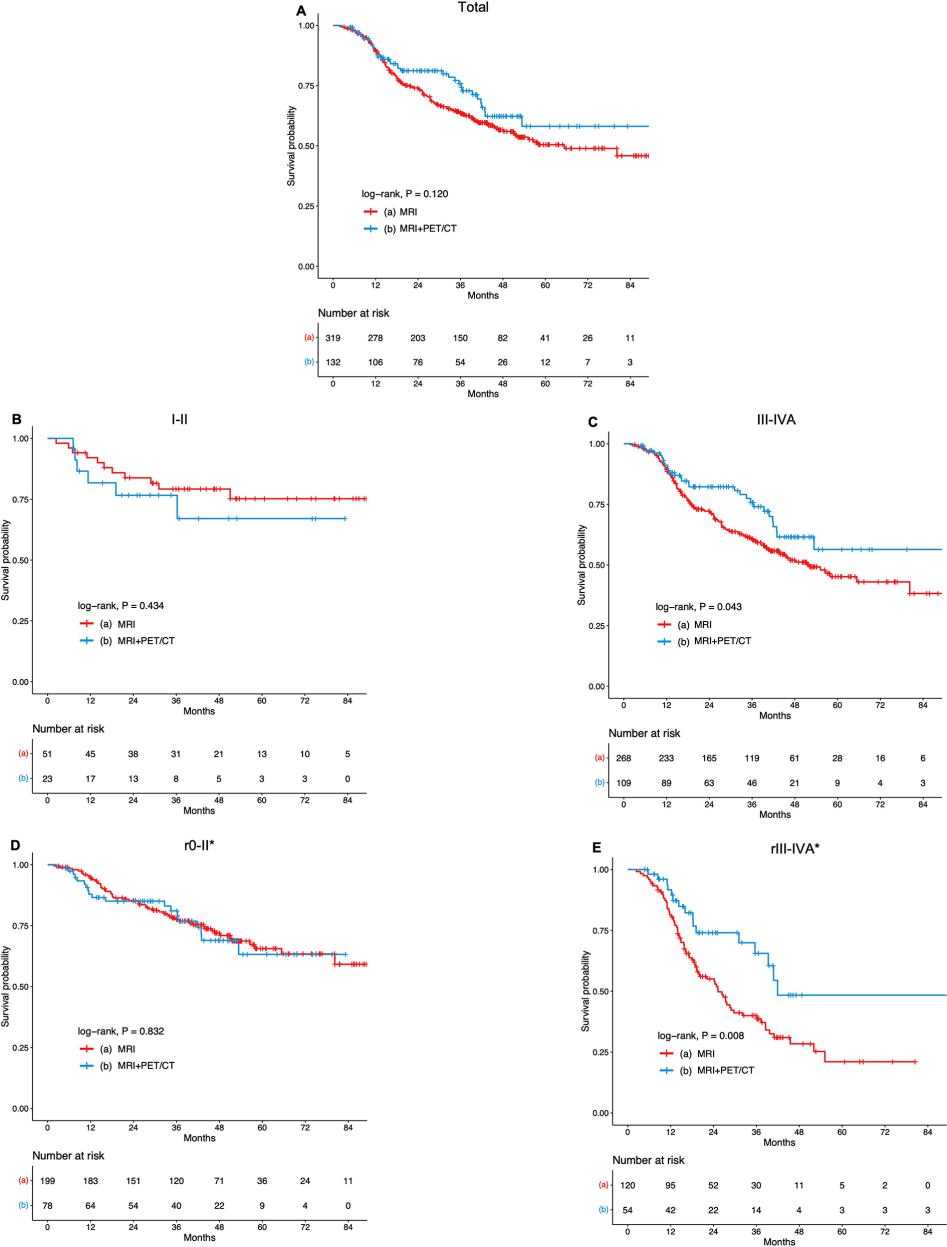
Kaplan–Meier curves of overall survival for NPC patients stratified by the MRI and with PET/CT groups: (**A**) Total; (**B**) I-II; (**C**) III-IVA; (**D**) r0-II; (**E**) rIII-IVA. Abbreviations: NPC: nasopharyngeal carcinoma; r: Recurrent; MRI: magnetic resonance imaging; PET/CT: positron emission tomography/computed tomography. *Based on the eighth edition of the Union for International Cancer Control/American Joint Committee on Cancer (UICC/AJCC) staging system according to MRI

When patients were assigned to MRI alone and with PET/CT groups, there was no significant difference between them in terms of OS (*P* = 0.120; Fig. 2A). However, subgroup analysis revealed that in the III-IVA (B cohort) NPC, patients who with PET/CT exhibited significantly higher 5-year OS rates (MRI vs. with PET/CT: 45.2% vs. 56.4%, *P* = 0.045) (Table S1; Fig. 2C). Conversely, in the I-II group, there was no significant difference in OS revealed by log-rank test (*P* = 0.434; Fig. 2B); Similarly, significant difference in 5-year OS was also observed in the rIII-IVA (C cohort) patients (MRI: 21.0% vs. with PET/CT: 48.4%; *P* = 0.009), but not in rI-II cohort (*P* = 0.832; Table S1; Fig. 2D, E). Then, we excluded cases with censored data in B and C cohorts and conducted a multivariate analysis including rEBV DNA, rCRP, rALB, rNLR, retreatment strategies, and PET/CT in the III-IVA and rIII-IVA groups. The results demonstrated that with PET/CT was an independent predictor of OS in the rIII-IVA category (HR = 0.476; 95% CI: 0.267 to 0.847; *P* = 0.012), but not in the the III-IVA group (HR = 0.731; 95% CI: 0.459 to 1.165; *P* = 0.188) (Table 2; Table S2). Subsequently, to further investigate the value of PET/CT, a subset of 121 cases with complete data and maximum standardized uptake value (SUVmax) in Cohort D was selected for analysis. In the univariate analysis, it was found that patients classified as r0-rII (based on PET/CT) and SUVmax < 11.9 indicated better OS rates (*P* = 0.023; *P* = 0.015, respectively; Table 3).

**Table 2:** Univariate and multivariate Cox regression analysis for overall survival in the rIII-IVA* NPC

Characteristics	Univariable analysis	*P*	Multivariate analysis	*P*
	HR (95% CI)		HR (95% CI)	
Age (years)				
< 45	1 (reference)			
≥ 45	1.090 (0.685–1.733)	0.717		
Sex				
Male	1 (reference)			
Female	1.372 (0.775–2.428)	0.278		
rEBV DNA (copies/ml)				
Undetectable	1 (reference)			
Detectable	1.612 (0.899–2.889)	0.109		
Unkown				
rLactate dehydrogenase (U/L)				
< 240	1 (reference)			
≥ 240	1.104 (0.347–3.518)	0.867		
Unkown	/			
rC-reactive protein (mg/L)				
< 10.5	1 (reference)		1 (reference)	
≥ 10.5	2.784 (1.615–4.798)	**< 0.001**	2.348 (1.336–4.127)	**0.003**
rAlbumin (g/L)				
< 39.4	1 (reference)		1 (reference)	
≥ 39.4	0.195 (0.107–0.356)	**< 0.001**	0.224 (0.120–0.418)	**< 0.001**
rNLR				
< 3.7	1 (reference)			
≥ 3.7	1.372 (0.855–2.203)	0.190		
Unkown	/			
Treatment				
Palliative treatment	1 (reference)		1 (reference)	
Re-radiotherapy + Salvage Surgery	0.340 (0.210–0.551)	**< 0.001**	0.407 (0.249–0.666)	**< 0.001**
PET/CT				
No	1 (reference)		1 (reference)	
Yes	0.519 (0.294–0.917)	**0.024**	0.476 (0.267–0.847)	**0.012**

Abbreviations: r: Recurrent; EBV: epstein-barr virus; NLR: neutrophil-to-lymphocyte ratio; HR: hazard ratio; 95% CI: 95% confidence interval; NPC: nasopharyngeal carcinoma; MRI: magnetic resonance imaging; PET/CT: positron emission tomography/computed tomography

* Based on the eighth edition of the Union for International Cancer Control/American Joint Committee on Cancer (UICC/AJCC) staging system according to MRI

Using the Cox regression model to calculate HR and 95% CI

**Table 3:** Univariate and multivariate Cox regression analysis for overall survival in the with PET/CT NPC cohort

Characteristics	Univariable analysis	*P*	Multivariate analysis	*P*
	HR (95% CI)		HR (95% CI)	
Age (years)		**0.034**		**0.040**
< 45	1 (reference)		1 (reference)	
≥ 45	2.277 (1.064–4.873)		2.293 (1.037–5.074)	
Sex		0.964		
Male	1 (reference)			
Female	0.978 (0.373–2.562)			
rEBV DNA (copies/ml)		0.691		
Undetectable	1 (reference)			
Detectable	1.165 (0.548–2.475)			
rLactate dehydrogenase (U/L)		0.620		
< 240	1 (reference)			
≥ 240	0.603 (0.082–4.446)			
rC-reactive protein (mg/L)		**< 0.001**		**0.003**
< 10.5	1 (reference)		1 (reference)	
≥ 10.5	4.465 (2.064–9.660)		3.552 (1.560–8.087)	
rNLR		0.344		
< 3.7	1 (reference)			
≥ 3.7	1.445 (0.675–3.095)			
rT category^#^		0.062		
rT0-2	1 (reference)			
rT3-4	2.122 (0.962–4.683)			
rN category^#^		0.372		
rN0	1 (reference)			
rN1-3	0.716 (0.344–1.49)			
rStage^#^		0.133		
r0-rII	1 (reference)			
rIII-rIVA	1.745 (0.843–3.608)			
rT category*		**0.010**		
rT0-2	1 (reference)			
rT3-4	2.684 (1.263–5.703)			
rN category*		0.701		
rN0	1 (reference)			
rN1-3	0.868 (0.423–1.784)			
rStage*		**0.023**		**0.036**
r0-rII	1 (reference)		1 (reference)	
rIII-rIVA	2.600 (1.143–5.916)		2.662 (1.066–6.649)	
Treatment		**0.019**		0.316
Palliative treatment	1 (reference)		1 (reference)	
Re-radiotherapy + Salvage Surgery	0.379 (0.168–0.853)		0.653 (0.284–1.502)	
PET/CT SUVmax		**0.015**		0.179
< 11.9	1 (reference)		1 (reference)	
≥ 11.9	2.464 (1.195–5.078)		1.730 (0.777–3.852)	

Abbreviations: r: Recurrent; EBV: epstein-barr virus; NLR: neutrophil-to-lymphocyte ratio; HR: hazard ratio; 95% CI: 95% confidence interval; NPC: nasopharyngeal carcinoma; MRI: magnetic resonance imaging; PET/CT: positron emission tomography/computed tomography; SUVmax: maximum standardized uptake value

# Based on the eighth edition of the Union for International Cancer Control/American Joint Committee on Cancer (UICC/AJCC) staging system according to MRI

* Based on the eighth edition of the Union for International Cancer Control/American Joint Committee on Cancer (UICC/AJCC) staging system according to PET/CT

Using the Cox regression model to calculate HR and 95% CI

### With PET/CT vs. MRI alone in staging

**Fig. 3:**
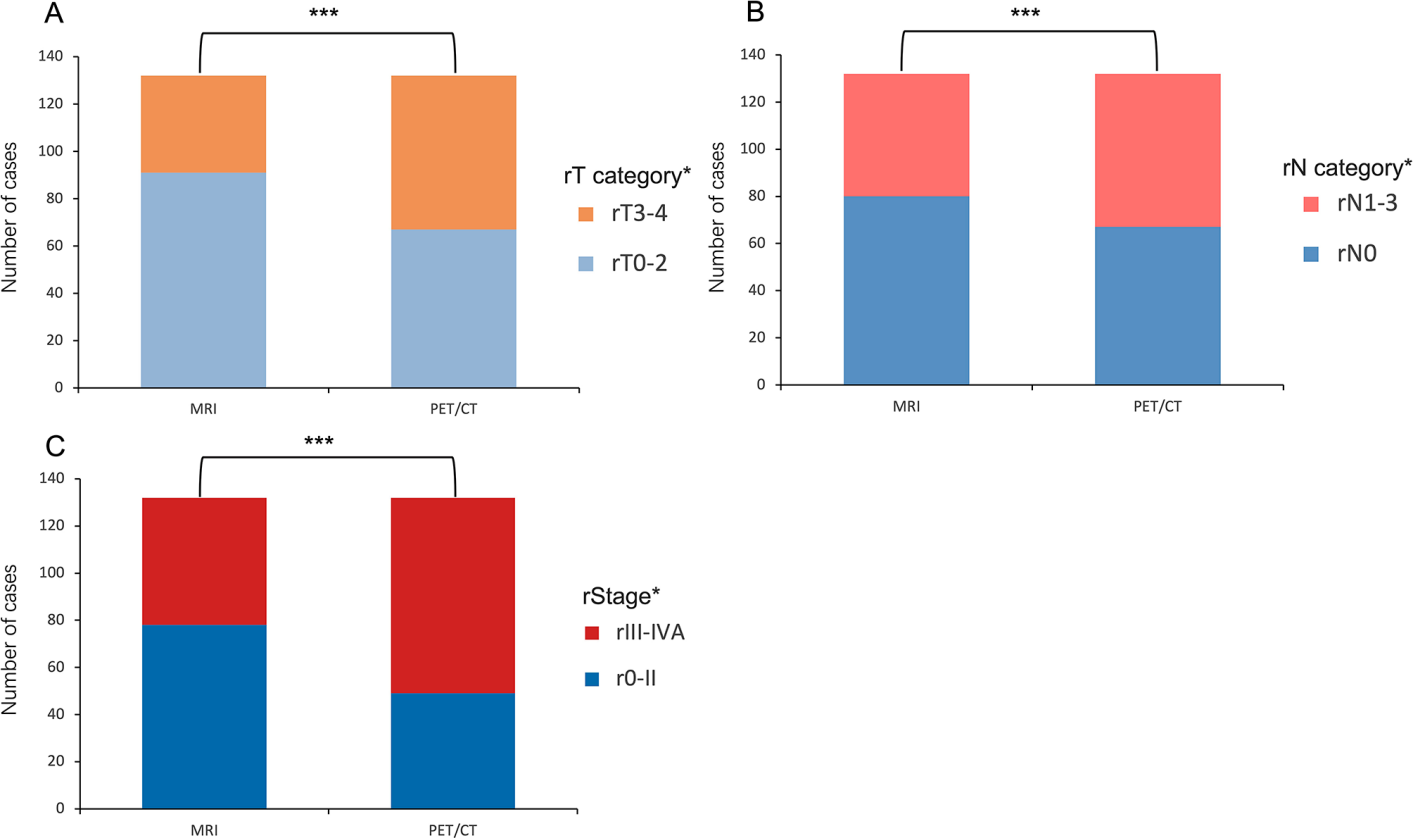
Comparison of the different stages of rT category(**A**), rN category(**B**), and rStage(**C**) for locoregional recurrent nasopharyngeal carcinoma staged by MRI and PET/CT. Abbreviations: r: Recurrent; NPC: nasopharyngeal carcinoma; MRI: magnetic resonance imaging; PET/CT: positron emission tomography/computed tomography. *Based on the eighth edition of the Union for International Cancer Control/American Joint Committee on Cancer (UICC/AJCC) staging system. *P* values were calculated using the Chi-squared test, ***P < 0.001

In cohort D, all patients underwent PET/CT and MRI scans. The rStage evaluated by MRI was r0 ([30/132] 22.7%), rI ([13/132] 9.8%), rII ([35/132] 26.5%), rIII ([24/132] 18.2%), and rIVA ([30/132] 22.7%) (Table S4; Fig. 3). In contrast, PET/CT resulted in a different distribution of rStage, with r0 ([4/132] 3%), rI ([9/132] 6.8%), rII ([36/132] 27.3%), rIII ([44/132] 33.3%), and rIVA ([39/132] 29.5%) (Table S4; Fig. 3). These results suggest that undergoing PET/CT is more likely to lead to an upstaging of rStage compared to MRI. Moreover, Using PET/CT sacns, rStage was identified as an independent predictor of OS (r0-rII vs. rIII-IVA; HR = 0.376; 95% CI: 0.150 to 0.938; *P* = 0.036) (Table 3); there were no significant differences in OS between those who underwent MRI (Table 3).

### Treatment modified based on PET/CT

**Fig. 4:**
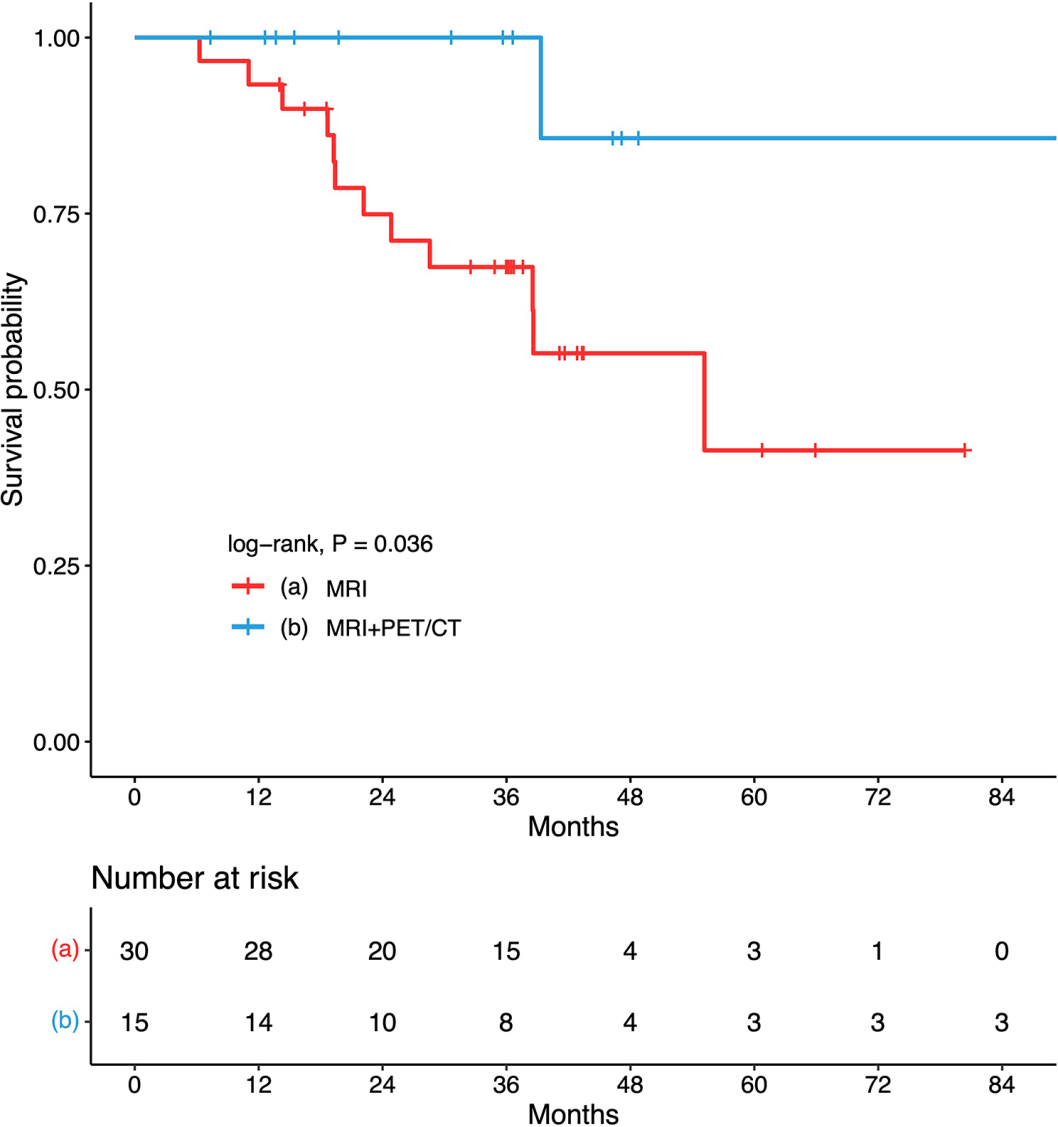
Kaplan–Meier curves of overall survival for the rIII-IVA NPC patients with salvage surgery stratified by the MRI and with PET/CT groups. Abbreviations: r: Recurrent; MRI: magnetic resonance imaging; NPC: nasopharyngeal carcinoma; PET/CT: positron emission tomography/computed tomography

There were no significant statistical differences in terms of treatment strategies between the MRI alone and with PET/CT groups in the rIII-IVA and III-IVA NPC, as indicated in Supplementary Table S3 and Figure S2. However, there was a higher tendency for patients in the rIII-IVA and III-IVA groups who underwent PET/CT to receive surgery, while a smaller proportion received palliative treatment compared to patients in the r0-II and I-II stages (Fig. S3). In other words, the application of PET/CT increased the likelihood of patients receiving surgery. Furthermore, in the rIII-IVA NPC, patients receiving PET/CT sacns before salvage surgery had a better prognosis compared with MRI alone (*P* = 0.036) (Fig. 4 and Table S5).

## Discussion

The present study enrolled the recurrent NPC patients without distant metastasis to investigate the prognostic role of PET/CT. The findings demonstrated that with PET/CT was an independent predictor of OS in lrNPC patients with stage rIII-IVA disease (HR = 0.476; *P* = 0.012; Table 2). Using PET/CT sacns, rStage was identified as an independent predictor of OS (r0-rII vs. rIII-IVA; HR = 0.376; 95% CI: 0.150 to 0.938; *P* = 0.036) (Table 3). Additionally, in the rIII-IVA NPC, patients receiving PET/CT sacns before salvage surgery had a better prognosis (Fig. 4; Table S5).

Of note, numerous studies have demonstrated the superiority of PET/CT over MRI in diagnosing primary and/or regional recurrence and accurately staging the recurrence of NPC after IMRT [15–17]. The present study is the first attempt to investigate whether these advantages of PET/CT can translate into improved prognosis in lrNPC. A previous study found that the rT3-4N0M0 NPC staged by PET/CT plus MRI had a higher 3-year OS than patients staged using MRI alone (adjusted HR = 0.34; *P* = 0.005) [17]. In contrast, for NPC patients with III-IVA and rIII-IVA staged by MRI, we found that with PET/CT also resulted in a better prognosis compared to MRI alone (*P* = 0.045 and *P* = 0.009, respectively) (Table S1; Fig. 2C, E). Furthermore, with PET/CT was identified as an independent predictor of OS in the rIII-IVA groups (HR = 0.476; *P* = 0.012; Table 2). However, there was no statistically significant difference between MRI alone and with PET/CT for all lrNPC in terms of OS (*P* = 0.120; Fig. 2A). These results suggest that the inclusion of PET/CT is necessary, particularly for patients with recurrent stage III-IVA disease who have already been evaluated using MRI.

We attempted to investigate the reasons behind the improved prognosis associated with the use of PET/CT. There was no statistically significant difference in terms of treatment strategies between MRI alone and with PET/CT in the rIII-IVA NPC. However, there was a tendency for a greater proportion of patients to undergo re-radiotherapy with PET/CT, which might provide a survival benefit, as shown in Supplementary Table S3 and Figure S2. We also observed that the application of PET/CT increased the likelihood of patients undergoing surgery (Fig. S3). One of the possible reasons is that surgeons may be more willing to use PET/CT for preoperative evaluation to rule out distant metastases. Additionally, our further study found that preoperative PET/CT could improve prognosis in rIII-IVA patients undergoing salvage surgery compared with MRI alone, suggesting that PET/CT may also provide a more precise surgical scope. In general, more precise identification of tumors often leads to more precise radiotherapy. Huang et al. demonstrated that PET/CT parameters could accurately delineate the target tumor for NPC radiotherapy [21]. Altogether, we hypothesized that based on the results of PET/CT, patients may receive more accurate surgical coverage and precise radiotherapy, which could render the improved prognosis.

Currently, the recurrent TNM (rTNM) of the Union for International Cancer Control/American Joint Committee on Cancer (UICC/AJCC) staging system is still widely used to predict recurrent NPC prognosis [14, 22]. However, there are variations in outcomes among patients with lrNPC in the same stage, as determined by rTNM [22–24]. Notably, the majority of rTNM staging was based on MRI evaluation. Interestingly, Sun et al. developed a nomogram that incorporated rT-stage (mostly evaluated by MRI) as well as other factors and demonstrated satisfactory performance in predicting OS for lrNPC [25]. Similarly, Wen et al. combined rT-stage (based on PET/CT) and other baseline parameters to construct a prediction model for lrNPC, which also achieved ideal discrimination for OS [26]. In our study, we identified rStage-PET/CT (based on PET/CT) was an independent predictor of OS. (r0-rII vs. rIII-IVA; HR = 0.376; *P* = 0.036) (Table 3). While there was no statistically significant difference in terms of OS among rStage based on MRI scans (Table 3). What’s more, consistent with previous studies, we also found that with PET/CT was more likely to result in upstaging of rStage compared to MRI (Table S3; Fig. 3) [17]. Therefore, based on the available evidence, PET/CT appears to be a more effective method than MRI for assessing recurrent TNM staging.

PET/CT parameters have been shown to provide valuable information about tumor metabolism, particularly the SUVmax of [18 F] FDG. Several studies have demonstrated that SUVmax is associated with prognosis in cancers [26–29]. Lin et al. revealed that a higher SUVmax of distant metastatic lesions was an unfavorable risk factors for OS (*P* = 0.005) in the metastatic NPC [28]. In de novo recurrent NPC patients, Yan et al. discovered the high SUVmax of metastatic lesions (≥ 10) at diagnosis independently predicted poor survival [27]. Our study found that SUVmax < 11.9 was associated with better 5-year OS rates (*P* = 0.011; Table 3) in the univariate analysis. However, it did not emerge as an independent predictor for OS in lrNPC. It is worth noting that these findings should be further explored in larger sample sizes or prospective studies to validate their significance.

Several shortcomings should be acknowledged. Firstly, the study design was retrospective, which may introduce selection bias. Therefore, further prospective studies are needed to validate the findings. Secondly, due to the limited sample size, the evaluation of PET/CT parameters across different treatment modalities might exist bias. Future studies can further investigate our findings in both endemic and non-endemic cohorts. Thirdly, other potential indicators derived from PET/CT, such as metabolic tumor volume (MTV) and total lesion glycolysis (TLG) can be adopted for prognosis prediction [26, 30]. Lastly, in present study, we adopted OS as the endpoint. Subsequent studies could consider using progression-free survival (PFS) as an endpoint indicator, which may provide more information on relapse associated with mortality.

## Conclusions

To our knowledge, this study represents the first confirmation that the utilization of PET/CT can enhance the survival outcomes of patients with the rIII-IVA NPC. Additionally, PET/CT appears to be a more effective method than MRI for assessing rTNM staging.

### Electronic supplementary material

Below is the link to the electronic supplementary material.

Supplementary Material 1
**Supplementary table S1** Univariate analysis for overall survival in the III-IVA* and rIII-IVA* NPC.

Supplementary Material 2
**Supplementary table S2**Univariate and multivariate Cox regression analysis for overall survival in the III-IVA* NPC.

Supplementary Material 3
**Supplementary table S3**Characteristics of the III-IVA* and rIII-rIVA* stage NPC in the MRI alone and with PET/CT groups.

Supplementary Material 4
**Supplementary table S4**Recurrent NPC staged by PET/CT and MRI.

Supplementary Material 5
**Supplementary table S5**Univariate analysis for overall survival in the recurrent NPC without distant metastasis.

Supplementary Material 1
**Supplementary fig. S1** Maximally selected rank statistics for identifying the optimal cut-off value of the rALB = 39.4(A), rCRP = 10.5(B), SUVmax = 11.9(C), and rNLR = 3.7(D). Abbreviations: r: Recurrent; ALB: Albumin; CRP: C-reactive protein; SUVmax: maximum standardized uptake value; NLR: neutrophil-to-lymphocyte ratio.

Supplementary Material 2
**Supplementary fig. S2** The proportions of salvage surgery, palliative treatment, and re-radiotherapy in the rIII-IVA NPC patients for the MRI and with PET/CT groups. Abbreviations: NS: no significance; MRI: magnetic resonance imaging; PET/CT: positron emission tomography/computed tomography.

Supplementary Material 3
**Supplementary fig. S3** The percentages on the vertical axis refer to the proportions of salvage surgery, palliative treatment, and re-radiotherapy in the with PET/CT group subtracted from the values in the MRI alone group. For III-IVA and rIII-IVA, there were higher percentages of salvage surgery and lesser palliative treatment. Abbreviations: r: Recurrent; MRI: magnetic resonance imaging; PET/CT: positron emission tomography/computed tomography.
Note: All the recurrent stages were based on the eighth edition of the Union for International Cancer Control/American Joint Committee on Cancer (UICC/AJCC) staging system according to MRI.

## Data Availability

The datasets generated and/or analyzed during the current study at the Sun Yat-sen University Cancer Center are not publicly available due to Chinese law and regulations but are available from the corresponding author on reasonable request.

## Declarations

### Abbreviations

FDG Fluorodeoxyglucose.

PET/CT Positron emission tomography/computed tomography.

lrNPC Locoregional recurrent nasopharyngeal.

r Recurrent/recurrence.

OS Overall survival.

HR Hazard ratio.

MRI Magnetic resonance imaging.

IMRT Intensity-modulated radiotherapy.

IC Induction chemotherapy.

VS Versus.

CT Computed tomography.

LDH Lactate dehydrogenase.

CRP C-reactive protein.

ALB Albumin.

EBV Epstein-Barr virus.

FSE Fast spin-echo.

TR Repetition time.

TE Echo time.

2D Two dimensions.

3D Three dimensions.

AUC Area under the curve.

ROC Receiver operating characteristic.

IQR Interquartile range.

SUVmax Maximum standardized uptake value.

UICC/AJCC Union for International Cancer Control/American Joint Committee. on Cancer.

MTV Metabolic tumor volume.

TLG Total lesion glycolysis.
