# Correction: Prognostic value of pre-treatment [^18^F] FDG PET/CT in recurrent nasopharyngeal carcinoma without distant metastasis

**DOI:** 10.1186/s12885-024-12276-9

**Published:** 2024-04-25

**Authors:** Zhe Dong, Gao-Yuan Wang, Dong-Yu Dai, Guan-Jie Qin, Ling-Long Tang, Cheng Xu, Jun Ma

**Affiliations:** Department of Radiation Oncology, State Key Laboratory of Oncology in South China, Guangdong Key Laboratory of Nasopharyngeal Carcinoma Diagnosis and Therapy, Guangdong Provincial Clinical Research Center for Cancer, Sun Yat-sen University Cancer Center, Guangzhou, 510060 P.R. China

**Correction to: BMC Cancer 24, 466 (2024)**

10.1186/s12885-024-12189-7

Following publication of the original article [1], the author reported a typesetting error, whereby Tables 1, 2 and 3 were erroneously omitted from the published version. The publishers apologise for this error. The omitted Tables 1, 2 and 3 are given below. The original article [1] has been corrected.

**Table 1:** Patient characteristics

Characteristics	With PET/CT *N*=132 (%)	Without PET/CT *N*=319 (%)
Age (years)		
< 45	65 (49.2%)	169 (53.0%)
≥ 45	67 (50.8%)	150 (47.0%)
Sex		
Male	106 (80.3%)	238 (74.6%)
Female	26 (19.7%)	81 (25.4%)
rEBV DNA (copies/ml)		
Undetectable	48 (36.4%)	105 (32.9%)
Detectable	69 (52.3%)	167 (52.4%)
Unkown	15 (11.4%)	47 (14.7%)
rLactate dehydrogenase (U/L)		
< 240	117 (88.6%)	247 (77.4%)
≥ 240	5 (3.8%)	7 (2.2%)
Unkown	10 (7.6%)	65 (20.4%)
rC-reactive protein (mg/L)		
< 10.5	107 (81.1%)	218 (68.3%)
≥ 10.5	18 (13.6%)	30 (9.4%)
Unkown	7 (5.3%)	71 (22.3%)
rAlbumin (g/L)		
<39.4	4 (3.0%)	30 (9.4%)
≥ 39.4	121 (91.7%)	224 (70.2%)
Unkown	7 (5.3%)	65 (20.4%)
rNLR		
< 3.7	89 (67.4%)	189 (59.2%)
≥ 3.7	41 (31.1%)	108 (33.9%)
Unkown	2 (1.5%)	22 (6.9%)
rT category*		
rT0-rT2	91 (68.9%)	228 (71.5%)
rT3-rT4	41 (31.1%)	91 (28.5%)
rN category*		
rN0	80 (60.6%)	159 (49.8%)
rN1-rN3	52 (39.4%)	160 (50.2%)
rStage*		
r0-rII	78 (59.1%)	199 (62.4%)
rIII-rIVA	54 (40.9%)	120 (37.6%)
Intial stage		
I-II	23 (17.4%)	51 (16.0%)
III-IVA	109 (82.6%)	268 (84.0%)
Pathological examination sites		
Primary tumor	79 (59.8%)	172 (53.9%)
Regional lymph nodes	50 (37.9%)	138 (43.3%)
Both	3 (2.3%)	9 (2.8%)
Treatment		
Salvage Surgery	60 (45.5%)	160 (50.2%)
Re-radiotherapy	48 (36.4%)	84 (26.3%)
Palliative treatment	24 (18.2%)	75 (23.5%)

*Abbreviations* r: Recurrent; EBV: epstein-barr virus; NLR: neutrophil-to-lymphocyte ratio; PET/CT: positron emission tomography/computed tomography

*Based on the eighth edition of the Union for International Cancer Control/American Joint Committee on Cancer (UICC/AJCC) according to MRI

**Table 2:** Univariate and multivariate Cox regression analysis for overall survival in the rIII-IVA* NPC

Characteristics	Univariable analysis	P	Multivariate analysis	P
	HR (95% CI)		HR (95% CI)	
Age (years)				
< 45	1 (reference)			
≥ 45	1.090 (0.685-1.733)	0.717		
Sex				
Male	1 (reference)			
Female	1.372 (0.775-2.428)	0.278		
rEBV DNA (copies/ml)				
Undetectable	1 (reference)			
Detectable	1.612 (0.899-2.889)	0.109		
Unkown				
rLactate dehydrogenase (U/L)				
< 240	1 (reference)			
≥ 240	1.104 (0.347-3.518)	0.867		
Unkown	/			
rC-reactive protein (mg/L)				
< 10.5	1 (reference)		1 (reference)	
≥ 10.5	2.784 (1.615-4.798)	**<0.001**	2.348 (1.336-4.127)	**0.003**
rAlbumin (g/L)				
< 39.4	1 (reference)		1 (reference)	
≥ 39.4	0.195 (0.107-0.356)	**<0.001**	0.224 (0.120-0.418)	**<0.001**
rNLR				
< 3.7	1 (reference)			
≥ 3.7	1.372 (0.855-2.203)	0.190		
Unkown	/			
Treatment				
Palliative treatment	1 (reference)		1 (reference)	
Re-radiotherapy+Salvage Surgery	0.340 (0.210-0.551)	**<0.001**	0.407 (0.249-0.666)	**<0.001**
PET/CT				
No	1 (reference)		1 (reference)	
Yes	0.519 (0.294-0.917)	**0.024**	0.476 (0.267-0.847)	**0.012**

*Abbreviations* r: Recurrent; EBV: epstein-barr virus; NLR: neutrophil-to-lymphocyte ratio; HR: hazard ratio; 95% CI: 95% confidence interval; NPC: nasopharyngeal carcinoma; MRI: magnetic resonance imaging; PET/CT: positron emission tomography/computed tomography

*Based on the eighth edition of the Union for International Cancer Control/American Joint Committee on Cancer (UICC/AJCC) staging system according to MRI

Using the Cox regression model to calculate HR and 95% CI

**Table 3:** Univariate and multivariate Cox regression analysis for overall survival in the with PET/CT NPC cohort

Characteristics	Univariable analysis	P	Multivariate analysis	P
	HR (95% CI)		HR (95% CI)	
Age (years)		**0.034**		**0.040**
< 45	1 (reference)		1 (reference)	
≥ 45	2.277 (1.064-4.873)		2.293 (1.037-5.074)	
Sex		0.964		
Male	1 (reference)			
Female	0.978 (0.373-2.562)			
rEBV DNA (copies/ml)		0.691		
Undetectable	1 (reference)			
Detectable	1.165 (0.548-2.475)			
rLactate dehydrogenase (U/L)		0.620		
< 240	1 (reference)			
≥ 240	0.603 (0.082-4.446)			
rC-reactive protein (mg/L)		**< 0.001**		**0.003**
< 10.5	1 (reference)		1 (reference)	
≥ 10.5	4.465 (2.064-9.660)		3.552 (1.560-8.087)	
rNLR		0.344		
< 3.7	1 (reference)			
≥ 3.7	1.445 (0.675-3.095)			
rT category^#^		0.062		
rT0-2	1 (reference)			
rT3-4	2.122 (0.962-4.683)			
rN category^#^		0.372		
rN0	1 (reference)			
rN1-3	0.716 (0.344-1.49)			
rStage^#^		0.133		
r0-rII	1 (reference)			
rIII-rIVA	1.745 (0.843-3.608)			
rT category*		**0.010**		
rT0-2	1 (reference)			
rT3-4	2.684 (1.263-5.703)			
rN category*		0.701		
rN0	1 (reference)			
rN1-3	0.868 (0.423-1.784)			
rStage*		**0.023**		**0.036**
r0-rII	1 (reference)		1 (reference)	
rIII-rIVA	2.600 (1.143-5.916)		2.662 (1.066-6.649)	
Treatment		**0.019**		0.316
Palliative treatment	1 (reference)		1 (reference)	
Re-radiotherapy+Salvage Surgery	0.379 (0.168-0.853)		0.653 (0.284-1.502)	
PET/CT SUVmax		**0.015**		0.179
< 11.9	1 (reference)		1 (reference)	
≥11.9	2.464 (1.195-5.078)		1.730 (0.777-3.852)	

*Abbreviations* r: Recurrent; EBV: epstein-barr virus; NLR: neutrophil-to-lymphocyte ratio; HR: hazard ratio; 95% CI: 95% confidence interval; NPC: nasopharyngeal carcinoma; MRI: magnetic resonance imaging; PET/CT: positron emission tomography/computed tomography; SUVmax: maximum standardized uptake value

#Based on the eighth edition of the Union for International Cancer Control/American Joint Committee on Cancer (UICC/AJCC) staging system according to MRI

*Based on the eighth edition of the Union for International Cancer Control/American Joint Committee on Cancer (UICC/AJCC) staging system according to PET/CT

Using the Cox regression model to calculate HR and 95% CI
