# Supplementary figures and images for: Prognostic value of pre-treatment [18F] FDG PET/CT in recurrent nasopharyngeal carcinoma without distant metastasis
Source: BMC Cancer. 2024 Apr 15;24:466. doi: 10.1186/s12885-024-12189-7 (PMC11017658; doi:10.1186/s12885-024-12189-7)

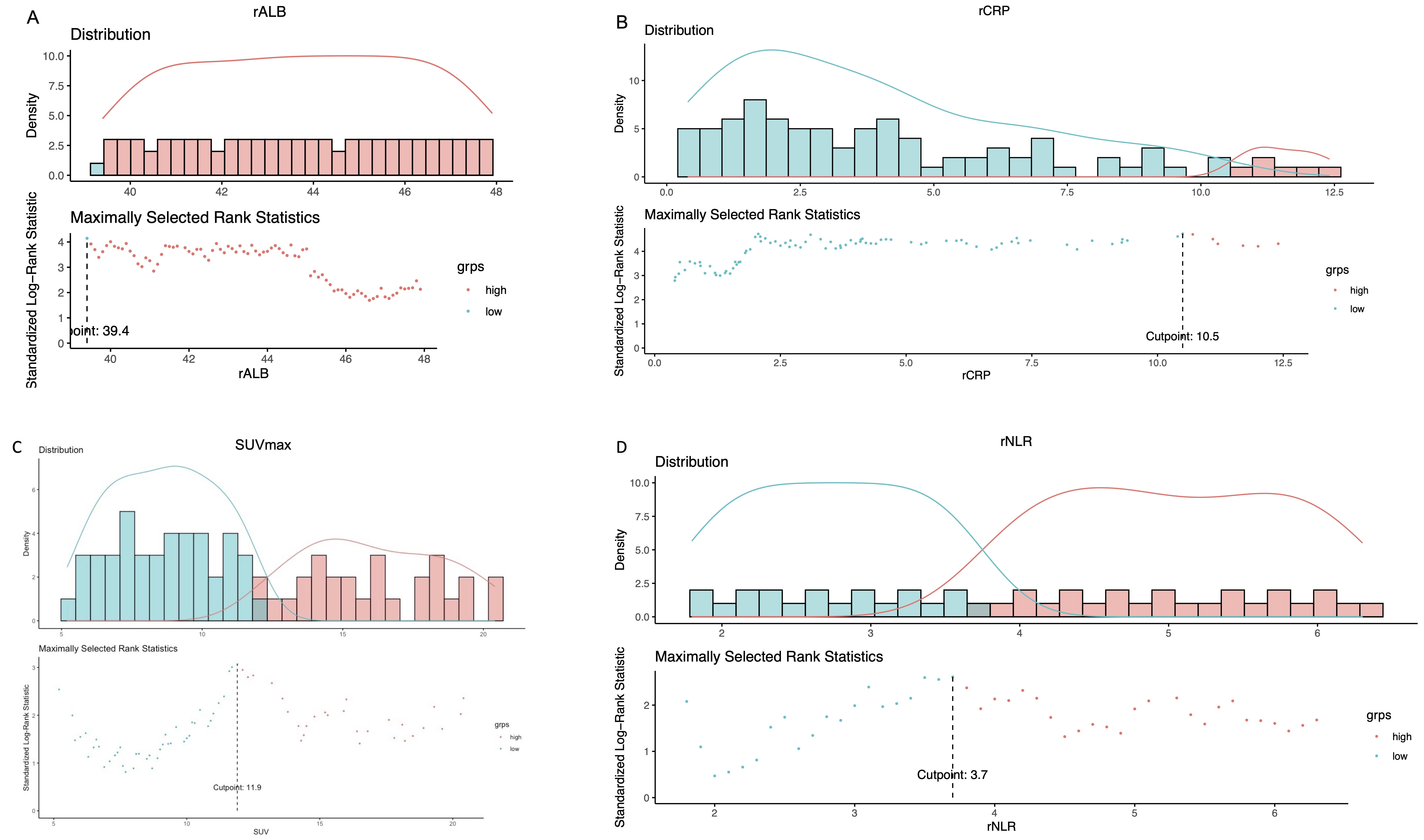

Supplement: Supplementary file 6 — Supplementary Material 1 Supplementary fig. S1 Maximally selected rank statistics for identifying the optimal cut-off value of the rALB = 39.4(A), rCRP = 10.5(B), SUVmax = 11.9(C), and rNLR = 3.7(D). Abbreviations: r: Recurrent; ALB: Albumin; CRP: C-reactive protein; SUVmax: maximum standardized uptake value; NLR: neutrophil-to-lymphocyte ratio. [file 12885_2024_12189_MOESM6_ESM.jpg]

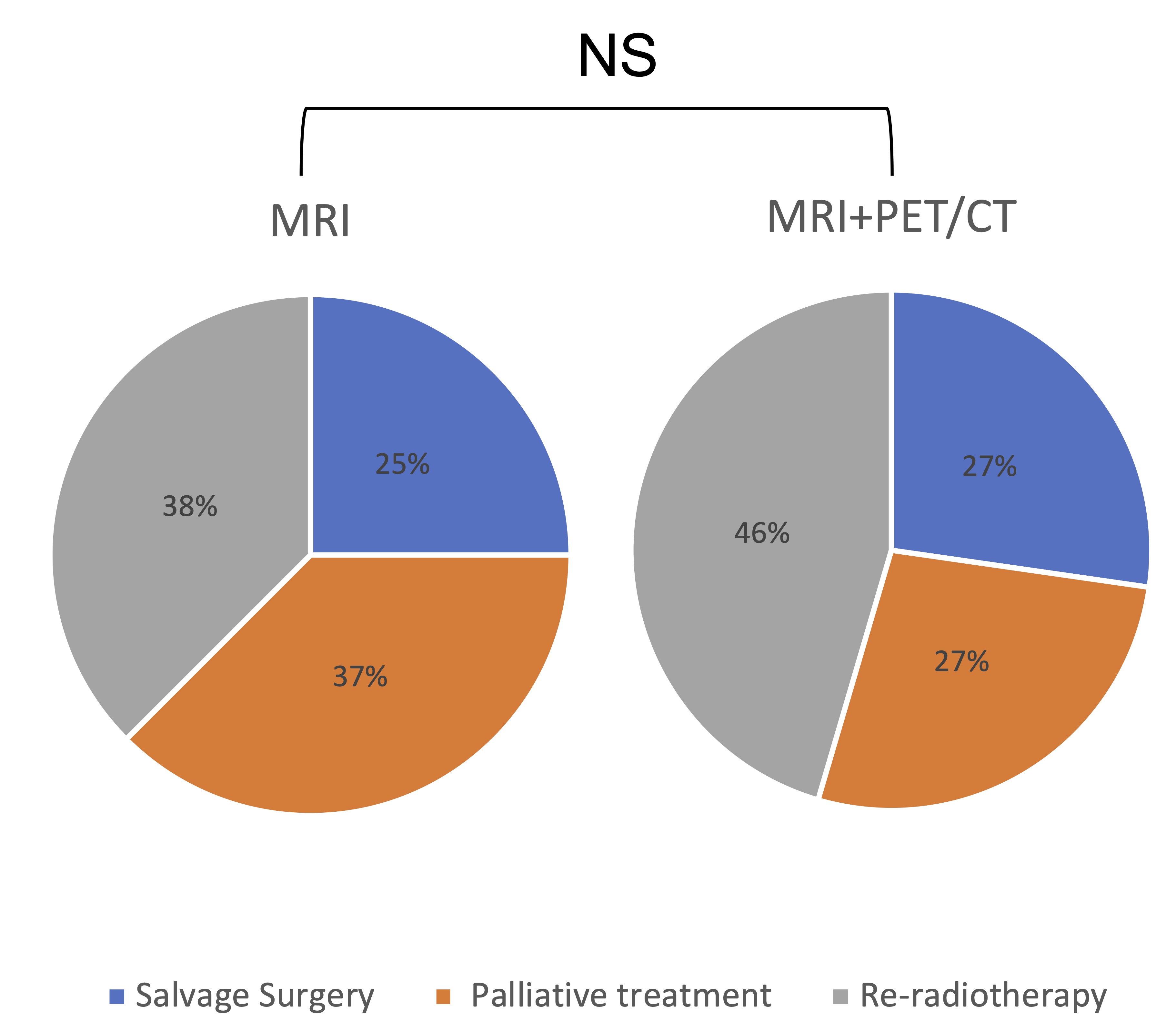

Supplement: Supplementary file 7 — Supplementary Material 2 Supplementary fig. S2 The proportions of salvage surgery, palliative treatment, and re-radiotherapy in the rIII-IVA NPC patients for the MRI and with PET/CT groups. Abbreviations: NS: no significance; MRI: magnetic resonance imaging; PET/CT: positron emission tomography/computed tomography. [file 12885_2024_12189_MOESM7_ESM.jpg]

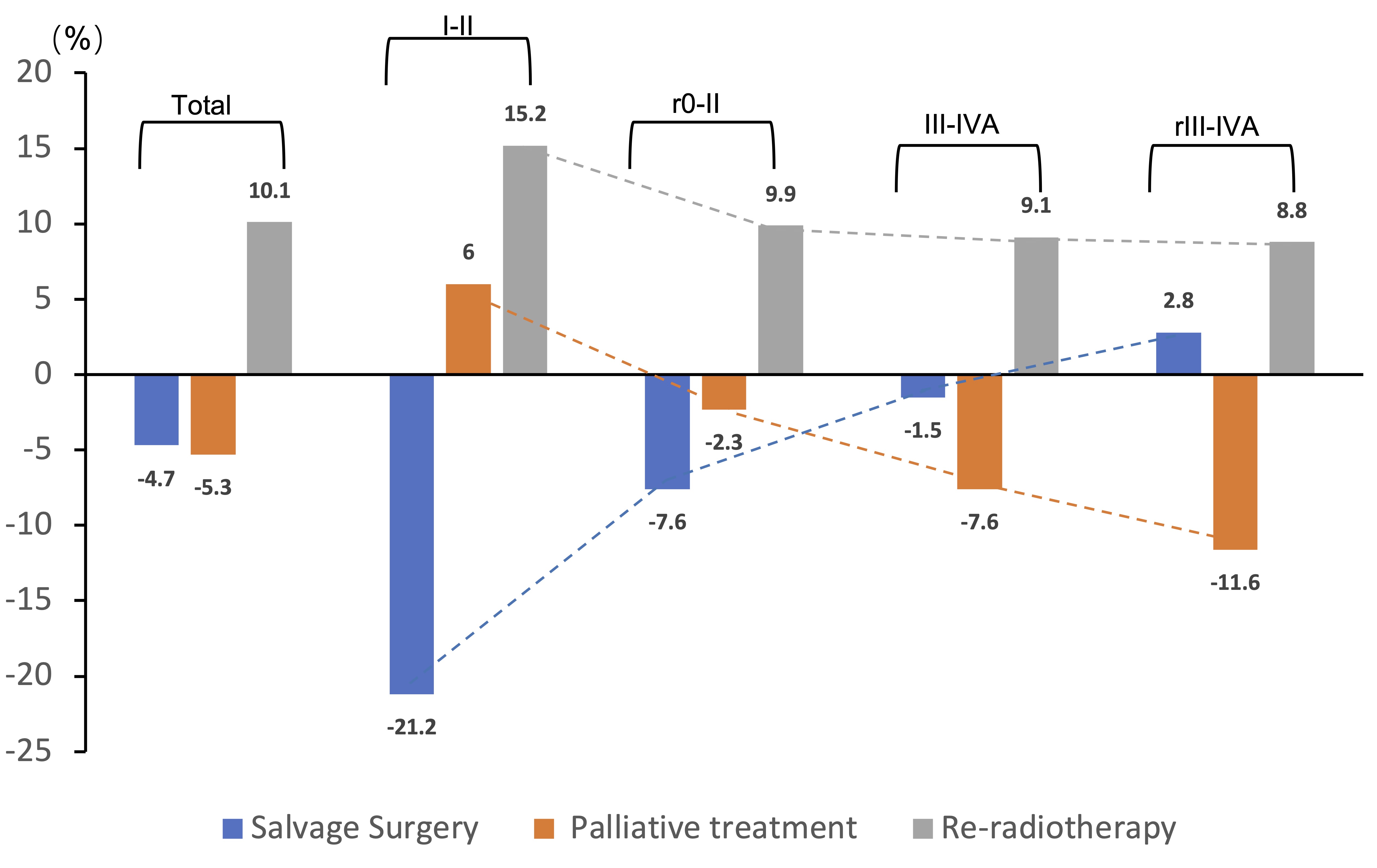

Supplement: Supplementary file 8 — Supplementary Material 3 Supplementary fig. S3 The percentages on the vertical axis refer to the proportions of salvage surgery, palliative treatment, and re-radiotherapy in the with PET/CT group subtracted from the values in the MRI alone group. For III-IVA and rIII-IVA, there were higher percentages of salvage surgery and lesser palliative treatment. Abbreviations: r: Recurrent; MRI: magnetic resonance imaging; PET/CT: positron emission tomography/computed tomography. Note: All the recurrent stages were based on the eighth edition of the Union for International Cancer Control/American Joint Committee on Cancer (UICC/AJCC) staging system according to MRI. [file 12885_2024_12189_MOESM8_ESM.jpg]
